# The Influence of Audio-Visual Interactions on Psychological Responses of Young People in Urban Green Areas: A Case Study in Two Parks in China

**DOI:** 10.3390/ijerph16101845

**Published:** 2019-05-24

**Authors:** Shilun Zhang, Xiaolong Zhao, Zixi Zeng, Xuan Qiu

**Affiliations:** 1 Key Laboratory of Cold Region Urban and Rural Human Settlement Environment Science and Technology, Ministry of Industry and Information Technology, School of Architecture, Harbin Institute of Technology, Harbin 150001, China; zixi_zeng@outlook.com (Z.Z.); qiux.x@outlook.com (X.Q.); 2 School of Architecture and Urban Planning, Suzhou University of Science and Technology, Suzhou 215009, China

**Keywords:** audio-visual interaction, audio-visual walk, young people’s psychological response, orthogonal analysis, urban parks

## Abstract

Audio-visual interactions in green spaces are important for mental health and wellbeing. However, the influence of audio-visual interactions on psychological responses is still less clear. This study introduced a new method, namely the audio-visual walk (AV-walk), to obtain data on the audio-visual context, audio-visual experiences, and psychological responses in two typical parks, namely Cloves Park and Music Park in Harbin, China. Some interesting results are as follows: First, based on Pearson’s correlation analysis, sound pressure level and roughness were significantly correlated with psychological responses in Cloves Park (*p* < 0.05). Second, the results of stepwise regression models showed the impact intensity of acoustic comfort was 1.64–1.68 times higher than that of visual comfort on psychological responses of emotion dimension, while visual comfort was 1.35–1.37 times higher than acoustic comfort on psychological responses of cognition dimension in Music Park. In addition, an orthogonal analysis diagram explained the influence of audio-visual interactions on psychological responses of young people. The audio-visual context located beside the waterscape with a relatively higher level of acoustic and visual comfort was the most cheerful (2.60), relaxed (2.45), and energetic (2.05), while the audio-visual context close to an urban built environment tended to be both acoustically and visually uncomfortable, and the psychological state was decreased to the most depressed (−0.25), anxious (−0.75), fatigued (−1.13) and distracted (−1.13).

## 1. Introduction

Psychological health issues of specific populations, especially young people, have developed into a major concern. People face a series of economic, academic, social, and life pressures that (without the possibility of recovery) result in vulnerability to stress, and ignoring psychological stress can lead to increased depression [1], diabetes [2], and cardiovascular and neurological illnesses [3]. On the other hand, urban green spaces are recognized as a context that can improve the psychological health of people because the environment in urban green spaces helps to reduce stress and assists with psychological recovery. Therefore, the context of urban green spaces is of importance.

Two theories, namely stress recovery theory (SRT) [4], which focuses on the recovery from stress due to attention to the contact with nature, and attention restoration theory (ART) [5], which focuses on recovery from mental fatigue using the natural environment, have been frequently used to explore the psychological restoration effects of the natural environment. Some previous studies employing SRT and ART explained the psychological restoration effects on the visual aspect perspective. Because watching "green nature" can lower the heartbeat and blood pressure and stimulate the parasympathetic nervous system, it calmed the sympathetic nervous system and increased the user’s tendency to relax and experience enjoyment and energy [6]. Much literature has described the application of different visual stimuli using visual stimulations from different open spaces (e.g., urban environment, parks, gardens, and forests) to compare psychological restoration effects in experimental research [7,8] or field studies [9,10,11]. Some other studies have quantified the visual landscape to explore the extent to which the visual landscape in green spaces can influence psychological responses. For example, a study by Grahn and Stigsdotter carried out in nine Swedish cities claimed that space openness and richness in the resident species, which was described by perceived sensory dimensions (PSDs), were significant factors that influenced the level of stress [12]. Han reported the results from studies in which the objective visual green rate was collected by a camera and analyzed with Photoshop and AutoCAD software. The results showed a significant correlation with fatigue nervousness and confusion reflected by a Profile of Mood State (POMS) in a natural environment on a university campus (*p* < 0.05) [11].

In recent years, some studies have assessed the psychological recovery potential of soundscapes [8,13,14,15]. They focused on the relationship between the acoustic environment and psychological responses [16,17] and they identified the sound sources that promoted positive emotions (stress recovery) [13,18]. According to a study by Daniel Shepherd et al., the mean scores of psychological well-being described by the WHO’s short-form quality of life instrument (WHOQOL-BREF) perceived by residents in quiet areas were higher than in noisy areas [19]. Based on the records of 180 subjects, Goel et al. showed that an auditory stimulus (birdsong enhanced with a classical music background at 60 dB) significantly reduced depression and anger reflected by a POMS after 15 min of exposure [20].

Accumulating evidence has shown that green space can reduce traffic-related annoyance via psychological mechanisms, including visual screening of the noise source and increased restorative quality of the residential environment [21,22]. Yang concluded, based on EEG-recordings, that visually-presented landscape vegetation can “provide excess noise attenuating effects through the subjects’ emotional processing” [23]. Thus, when exploring the effects of contextual factors on psychological responses, neither acoustic variables nor visual factors should be neglected, in particular, in green spaces. The importance of acoustic-visual interactions on both mental health and well-being was realized in a few studies [24]. For example, in two studies in the UK, Payne considered both visual and acoustic environments and they proposed that an urban soundscape was perceived as lower in restorative potential than an urban park soundscape, which was perceived as lower than that in rural areas [8]. Shu and Ma compared the effects of an urban park and classroom with 32 visual and auditory stimulation combinations (2 visual × 16 stimuli) on attention recovery of children using the Perceived Restorative Sounds Scale (PRSS) [25].

However, even though the relationship between audio-visual environment and psychological response has been studied in a qualitative way in many previous studies, the mechanism of audio-visual environment and audio-visual perception on the psychological response of users in urban parks still requires further improvement. In addition, the sound environment in an urban green space is complicated, because the green space contains multiple sound sources. However, many studies have focused on the effect of a single sound source. The visual variables analyzed in an audio-visual environment have also been too simplistic. Additionally, whether these psychological benefits could be generalized to young people has not been studied systematically since previous research primarily focused on adults [26,27], children [25,28] and the aged [29]. Urban parks in Europe have been seen as quiet areas that not only protect against noise but also reveal positive sounds [30]. Urban parks in Asia, however, especially in high-density cities in China, are impacted by high-level traffic noise. Thus, a study in urban parks in China is significant.

Two research questions were studied: 

RQ1: Do audio-visual contexts and experiences in urban parks influence psychological responses of young people?

RQ2: How do audio-visual interactions determine psychological responses of young people in urban parks?

## 2. Methods

### 2.1. Survey Sites

Two typical urban parks, namely Cloves Park and Music Park located in Harbin, China, were selected as survey sites. The reasons why these two urban parks were selected were the following: First, they are frequently visited by residents and tourists and are not far away from an urban center. Second, there are various kinds of landscapes (plants, mountains and lakes) and soundscapes (traffic sound, natural sounds, human activities sound, music and so forth) that enriched the sample. Third, the compositions of sound sources in these two parks may result in differences in psychological responses. In Cloves Park, traffic sounds, construction sounds, animal sounds (birdsong and insect songs), and activity sounds are dominant sounds that were investigated in a preliminary study. Traffic sounds, music, and activity sounds make up the main ingredients, while animal sounds can be heard in less 10% of the areas investigated in a preliminary survey in Music Park.

Previous studies adopted soundwalk methodologies for investigating the visual, acoustic or audio-visual environments on participants’ experience, especially acoustic perception in soundscape field [31,32]. Nevertheless, in this study, a new method named audio-visual walk (AV-walk) was used to obtain data on audio-visual contexts, audio-visual experiences, and psychological responses to explore the influence of audio-visual interactions on psychological responses of participants. In a preliminary study, a group (4 persons) that majored in Architecture Landscape was asked to visit these two parks and then mark the stopping locations that impressed them in terms of landscape and soundscape for formal research in each park. The choice of stopping locations is similar to soundwalk the method of Jeon et al. [31]. Then, according to the preference weight (>80%) of the stopping locations, we determined the final 30 survey points. In a formal study, participants were asked to walk on a pre-set path and when they visited one of 30 points in each park, they were asked to perceive the intensity of each sound source for 2–3 min [33], and to report the audio-visual experience and the psychological responses. At the same time, acoustic instruments and a camera were used to obtain data on the sound environment and visual environment, respectively.

The study was conducted in accordance with the Declaration of Helsinki, and the protocol was approved by the Research Ethics Committee of School of Architecture of Harbin Institute of Technology, China. In addition, all participants gave their informed consent for inclusion before the experiment of the audio-visual walk originally started.

### 2.2. Participants

A total of 36 graduate students (male: 16; female: 20) with self-reported normal vision and hearing [34,35] asserted that they can easily distinguish a certain sound in a complex sound environment, sense the strength of the sound, and recognize different colors from the School of Architecture, Harbin Institute of Technology, volunteered to join the AV-walk survey. The age of the participants ranged from 22–33 years old [36]. Before the formal survey, participants were asked have good sleep on the day before the survey and to study and work for 2–4 h on the survey day to reduce errors of psychological responses. In addition, when the formal survey began, to avoid disturbances among them during perceiving the audio-visual context, they were prohibited from communicating, eating, and walking. Participants were also required to provide their demographic characteristics, such as gender, age (22–25, 25–28, or 28–33 years old), and educational background (bachelor’s degree and master’s degree, and those reading for a master’s or doctoral degree) before the formal investigation.

### 2.3. Procedure

The survey was performed in the afternoon (from 1:30–3:30) with clear weather from September 20–October 20, 2018, which is the most vigorous season for vegetation growth and bird and insect activity. The range of the average air temperature, wind strength, and air quality indexes (PM 2.5 index [37]) during the experimental procedure was 18–21 °C, 3–4 degrees and 35–75 μg/m^3^, respectively. The formal survey consisted of two parts, including audio-visual context measurements and the audio-visual perception questionnaire.

Regarding the audio-visual context, the sound pressure level was recorded with a sound pressure level meter (type: BSWA801, BSWA TECH, Beijing, China) at every survey point. The sound pressure level meter was set to a slow-mode and A-weight, and a reading for instantaneous data was taken every 1 s. The probe of the sound-level meter was positioned more than 1.0 m away from any reflectors and more than 1.2 m off the ground [38]. A total of 3 min of data were obtained at each measurement position, and the corresponding A-weight equivalent sound pressure level (LAeq) was derived. To measure the psychoacoustic indicators, an acoustic recorder (type: SQuadriga Ⅱ, LANDTOP, Beijing, China) was used simultaneously with the BSWA 801. Artemis 12.0 software (LANDTOP, Beijing, China) was used to analyze the psychoacoustic variables; e.g., loudness (sone), roughness (asper) and sharpness (acum) [39]. The assessment of loudness was based on the N5 value rather than the average because it was more suitable for the evaluation of time-varying sounds [40]. In terms of visual context, based on the quantification of visual landscapes in photos, recent works explored the relationship between landscape attributes and landscape preferences [41,42] and showed the effects of landscape elements on aesthetic appreciation that may impact emotions. Thus, in this study, a single lens reflect camera (35-mm lens) was used to take a picture of a normal setting every 90° with a total of 3 pictures [11] covering a 180° view in the horizontal plane at a height of 1.7 m above the ground at each survey point [15]. The picture was used to obtain landscape elements (e.g., plants, sky and paving) identified by a volunteer. Some visual parameters; i.e., visual green rate (%), sky visibility and paving visibility, were used in this study to quantify these landscape elements. The visible greenness rate quantifies the amount of vegetation that is visible in the visual field and it is a more valid approach than the green cover ratio [11], which was processed and calculated with Photoshop 6.0 software (Adobe, San Jose, CA, USA) [9,43] according to the pixel weight in each photograph. The sky visibility and paving visibility were analyzed with the same process.

Audio-visual context measurement and application of the audio-visual perception questionnaire were performed simultaneously, which means when the volunteer collected data on the audio-visual context, participants were asked to perceive the audio-visual context. The audio-visual perception questionnaire included three parts. First, as participants visited each survey point, they were required to perceive the sound sources and then evaluate the intensity of each sound with a five-point Likert scale: 0, not at all; 1, a little; 2, moderately; 3, a lot; and 4, dominates completely [44]. Second, the participants were asked to assess the audio-visual experience at each location, including acoustic comfort and visual comfort using a 9-point bipolar scale that ranged from not at all to extremely (from −4–4). Third, this study assumed that there are always two dimensions of psychological states in urban green spaces, as assessed in previous studies; that is, positive and negative emotions. Eight adjectives, including 4 positive and 4 negative adjectives, were selected with high reliability and validity from the Profile of Mood State (POMS), the Positive and Negative Affect Scale (PANAS) and the Restoration Scale (RS), based on SRT and ART. In this paper, two dimensions, namely emotion and cognition dimensions, in psychological response were reflected by these adjectives. Among them, 4 adjectives—cheerful, depressed, relaxed and anxious—were used to evaluate the emotion dimension of psychological response [11,45], and other adjectives including energetic, fatigued, focused, and distracted were selected to assess the cognition dimension of psychological response [46]. Additionally, many studies have shown that a psychological assessment appears to be bipolar rather than unipolar or simply categorical. Therefore, these 8 adjectives were divided into 4 items, and each item reflected both positive and negative feelings, which were cheerful-depressed (CD), relaxed-anxious (RA), energetic-fatigued (EF), and focused-distracted (FD), respectively. A 9-point bipolar scale was used to evaluate these 4 items with values ranging from −4 (negative) to 4 (positive).

### 2.4. Data Statistics

SPSS software [47] was used to analyze the normality of samples, correlations, and regression of indicators in the two parks. First, the Kolmogorov-Smirnov test was used to analyze the normality of the collected data. The results showed that all of the *p* values were more than 0.05 (except for birdsong in Music Park: *p* = 0.008, and paving visibility in Cloves Park and Music Park: *p* = 0.014 and 0.013, respectively), which indicated all samples had statistical significance. This may be because the sample of birdsong was low: The percentage of birdsong was less than 5% in 80% of the survey points. Second, Pearson’s correlation was used to calculate the relationship between the audio-visual context and psychological responses, the relationship between the audio-visual experiences and the psychological responses; a T-test at *p* < 0.01 and *p* < 0.05 was used to test for significant differences. Third, a stepwise linear regression was used to establish the regression equations for audio-visual context and psychological responses, audio-visual experiences and the psychological responses. An ANOVA was used to test the significance of regression equations. In addition, the reliability and validity of the questionnaire were also tested. The reliability and validity was measured by Cronbach’s Alpha and Kaiser-Meyer-Olkin (KMO) respectively. The values of Cronbach’s Alpha were 0.684 and 0.785 and the scores of KMO were 0.803 and 0.788 in Cloves Park and Music Park, respectively, which means that the reliability and validity of the questionnaire were acceptable [48].

The results of Pearson’s correlation analysis between the sound sources and psychological responses showed that traffic sounds and construction sounds were negatively correlated with psychological responses, birdsong and insect songs, and activity sounds were positively correlated with psychological responses in Cloves Park. In Music Park, traffic sounds and activity sounds were negatively correlated with psychological responses, and birdsong and music were positively correlated with psychological responses, although there was a weak correlation between music and psychological responses and between birdsong and psychological responses, which may be because the proportion of birdsong and activity sounds was relatively lower than other sounds at each survey point. Thus, traffic sounds, construction sounds, and activity sounds (in Music Park) were classified as negative sounds and birdsong, insect sounds, music activity sounds (in Cloves Park) were classified as positive sounds. 

## 3. Results

### 3.1. Identification of Audio-Visual Context and Experiences

Pearson’s correlation results between the acoustic context and psychological responses, and between visual context and psychological responses in the two parks are shown in Table 1. The results showed that LAeq, loudness (except for CD), and roughness were significantly negatively correlated with psychological responses in Cloves Park, with correlation coefficient *R* ranging from −0.850 to −0.396. There was no correlation between sharpness and psychological responses according to significance level (Sig.). It is interesting to note that LAeq and psychoacoustic parameters were uncorrelated with psychological responses in Music Park (*p* > 0.05). One possible reason may be that the composition of positive and negative sounds in Music Park is not significantly different; that is, the ratio of positive and negative sounds was close to 1:1 (ranging from 0.9–1.1) at 46.7% survey points in Music Park. Therefore, objective acoustic parameters may not reflect changes in participants’ psychological responses. Regarding visual context, there was no relationship between the visual green rate and psychological responses and between sky visibility and psychological responses in two urban parks (except for a weak correlation between sky visibility and RA in Music Park with a correlation coefficient *R* that was only 0.366).

Table 1 also shows the relationship between audio experiences and psychological responses and between visual experiences and psychological responses in two urban parks. In Cloves Park, it can be seen that acoustic comfort and visual comfort showed a positive strong correlation with psychological responses (*p* < 0.01) with coefficient *R* ranging from 0.511–0.889. Audio-visual experiences were also positively correlated with psychological responses (*p* < 0.05) with coefficient *R* ranging from 0.665–0.873 in Music Park. It can be concluded that, with audio-visual comfort increasing, the level of psychological responses, including CD, RA, EF, and FD, also increased, which was consistent with a recent research result in Spain [17].

Stepwise regression models (confidence interval 95%) were established to determine the impact intensity of audio-visual contexts on psychological responses in Cloves Park (shown in Table 2). To decrease errors, the variance inflation factor (VIF) was calculated to test the collinearity among predictors (VIF < 10). It can be seen that only the LAeq can be used to examine CD, RA, EF and FD in Cloves Park. There were no objective acoustic and visual parameters that could determine psychological responses (except for sky visibility) in Music Park.

Table 3 describes the final results of the stepwise regression analysis in terms of the impact intensity of audio-visual experiences on psychological responses, including CD, RA, EF and FD in Cloves Park. The results of a stepwise regression analysis suggested that the regression model was significant and four psychological responses named CD, RA, EF and FD were determined by different predictors. In Cloves Park, CD was determined by acoustic and visual comfort, and acoustic comfort was in a dominant position regarding the influence on CD, with a beta weight of 0.660, compared to visual comfort (*p* < 0.01). RA and EF were also determined by acoustic and visual comfort, and the relative contribution of acoustic comfort was 2.37 and 2.64 times higher than visual comfort (*p* < 0.01), respectively, while FD was only explained by acoustic comfort (*p* < 0.01).

In Music Park (Table 4), psychological responses can be examined by both acoustic comfort and visual comfort (*p* < 0.05). The predictor with the highest contribution was acoustic comfort in the model for both CD and RA. The impact intensities of acoustic comfort on CD and RA were 1.68 and 1.64 times higher than that of visual comfort, respectively, while in the model for EF and FD, visual comfort was more significant than acoustic comfort by 1.37 and 1.35 times, respectively.

### 3.2. Influence of Audio-Visual Interactions on Psychological Responses

**Figure 1:**
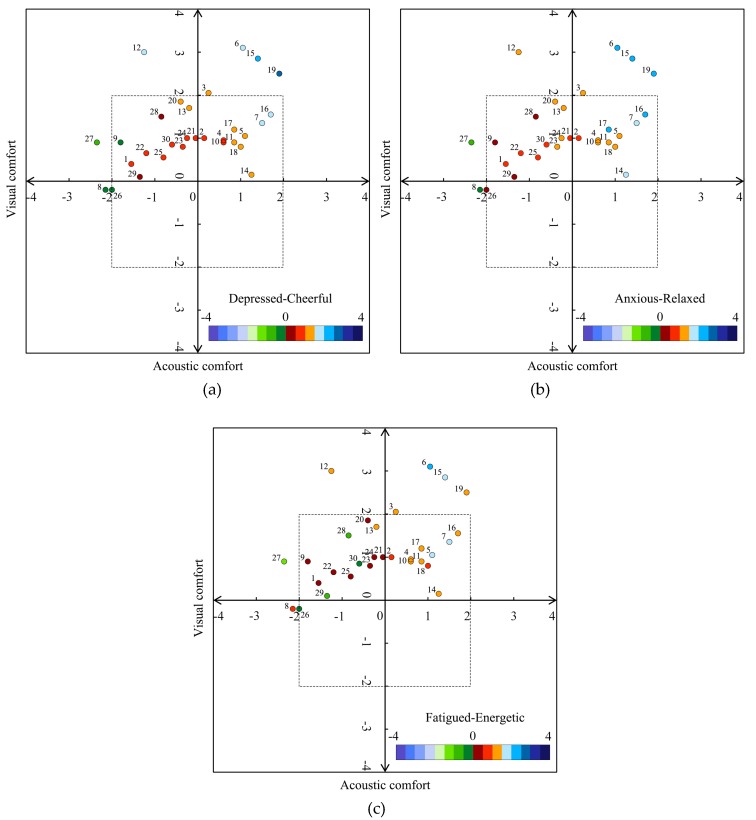
Orthogonal analysis of audio-visual experiences on psychological responses: (**a**) cheerful-depressed (CD); (**b**) relaxed-anxious (RA); (**c**) energetic-fatigued (EF) in Cloves Park.

**Figure 2:**
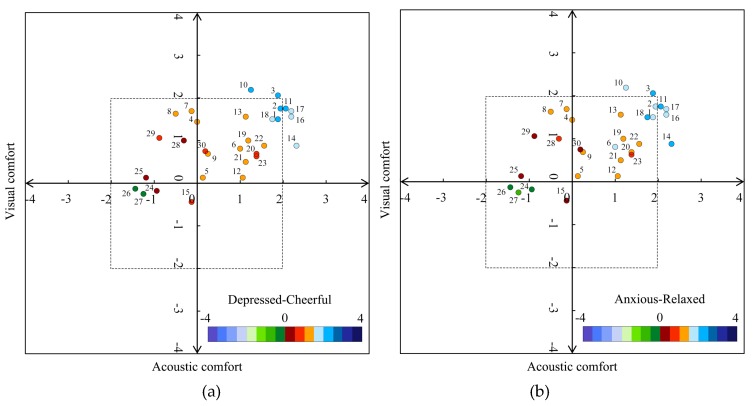

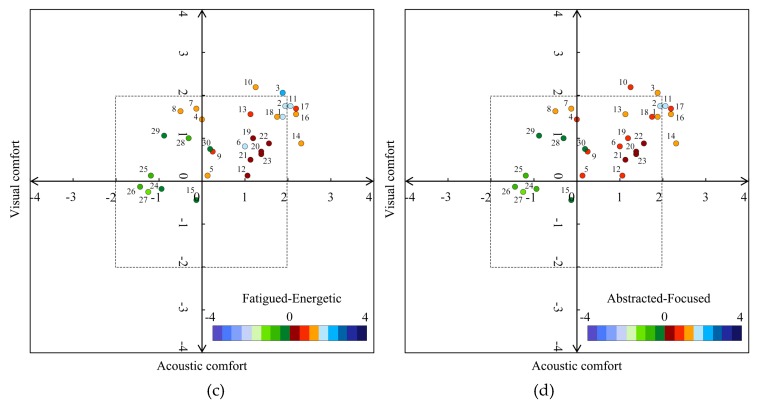
Orthogonal analysis of audio-visual experiences on psychological responses: (**a**) CD; (**b**) RA; (**c**) EF; (**d**) focused-distracted (FD) in Music Park.

The above results show the relationship between audio-visual contexts and psychological responses and between audio-visual experiences and psychological responses, and also analyzed the impact level of audio-visual independent variables on psychological responses in two urban parks. The results of multiple logical regressions showed the psychological health of young people in urban parks was determined by audio-visual experiences reflected by acoustic comfort and visual comfort (except for FD in Cloves Park). How audio-visual interactions determine psychological responses of young people in urban parks remains unclear. Thus, to answer this research question, an orthogonal analysis diagram was established to describe the influence of audio-visual interactions on psychological responses, shown in Figure 1 and Figure 2. The orthogonal analysis method was designed and used in this study to analyze the effects of visual and auditory factors that simultaneously affect the psychological changes acquired by participants instead of employing separate analyses. However, the orthogonal analysis model for FD was not established in this paper because FD cannot be examined by visual comfort. As illustrated in Figure 1 and Figure 2, psychological responses of every survey point were represented in a two-dimensional space defined by acoustic comfort and visual comfort. The positions for psychological responses in the two-dimensional space were determined by the values of acoustic comfort and visual comfort and the levels of psychological responses were represented by the color described in Figure 1 and Figure 2. To analyze the effects of audio-visual experiences in a readily comprehensible way, four quadrants were used to represent audio-visual interactions. The areas of quadrants 1, 2, 3, and 4 show the mean acoustic and visual comfortable (AV), acoustic uncomfortable and visual comfortable (UV), acoustic and visual uncomfortable (UU) and acoustic comfortable and visual uncomfortable (AU) results. 

In Figure 1, it can be seen that 46.7% of the survey points fell into the AV quadrant, 46.7% of the survey points fell into the UV quadrant, and 6.6% of the survey points fell into the UU quadrant for Cloves Park. Overall, 86.7% of the audio-visual context tended to be cheerful, 93.3% of the context tended to be relaxed, and 83.3% of the context was energetic. In the orthogonal analysis diagram of CD, the values of CD at 78.6% of the survey points were more than 1.00 in the AV quadrant, while the values of CD were more than 1.00 in 14.3% of the survey points in the UV quadrant. In terms of RA, the values of RA in 92.9% of the survey points were more than 1.00 in the AV quadrant, and the values of RA were more than 1.00 in 35.7% of the survey points in the UV quadrant. Regarding EF, the levels of EF in 85.7% of the audio-visual contexts were more than 1.00 in the AV quadrant, while the values of EF were more than 1.00 at 14.3% of the survey points in the UV quadrant.

**Figure 3:**
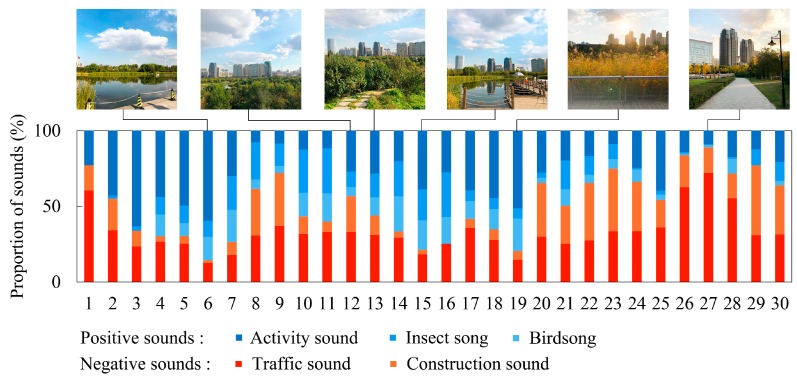
The proportion of sounds in each survey point and photographs in typical survey points in Cloves Park.

On the other hand, in the AV quadrant, the audio-visual context at survey point 19 was the most cheerful, with the level of CD at 2.60, compared to point 15 (2.40) and point 6 (1.95). Survey point 19 was also the most relaxed audio-visual context, compared to point 15 (2.35) and point 6 (2.25). In the orthogonal analysis diagram of EF, survey point 6 had the most energetic audio-visual context, with a value of EF at 2.05, compared to point 15 (1.80) and point 7 (1.65), and point 19 was close behind (1.45). Points 6, 15, and 19 were almost the most cheerful, relaxed and energetic contexts among all points, which is due to three points that were located by the lakeside with better landscapes than others and the percentage of positive sounds in these three points were also higher than others (Figure 3). Points 7 and 16 also had relatively cheerful (1.50, 1.95), relaxed (1.75, 2.10) and energetic (1.65, 1.45) contexts, although they were visually inferior to the above three places. In the UV quadrant, survey point 12 had the most cheerful (1.50) audio-visual context, and the audio-visual context at point 13 was most relaxed (1.30) and energetic (1.35). In fact, both points 12 and 13 had outstanding audio-visual contexts with higher values of CD, RA, and EF than the others, which is likely because they were located on the top of a mountain with unique visual experiences, although the acoustic comfort was negative. The most depressed, anxious and fatigued contexts were at point 27 in the UV quadrant, and the values of CD, RA, and EF at point 27 were even lower than those at points 8 and 26 in the UU quadrant. It can be explained by previous theories; namely, SRT and ART, that the percentages of urban sounds (like traffic noise and construction noise) and urban views were the most salient, and the memory of the daily stress of participants can easily be aroused by these stimuli.

In Music Park (Figure 2), it can be seen that 66.7% of the survey points fell into the AV quadrant, 20.0% of the survey points fell into the UV quadrant and 13.3% of the survey points fell into the UU quadrant in Music Park. Overall, 93.3% of the audio-visual context tended to be cheerful, 90.0% of the contexts tended to be relaxed, 73.3% of the contexts were energetic and focused. The values of CD, RA, and EF in 85.0%, 90.0%, and 55.0% of the survey points were more than 1.00 in the AV quadrant, respectively, and the values of CD, RA, and EF were more than 1.00 in only 30.0% of the survey points in the UV quadrant and UU quadrant. However, the value of FD was more than 1.00 in only 23.3% of the survey points in the AV quadrant, the value of FD was less than 0 in 70.0% of the survey points in the UV and UU quadrants. It was interesting to note that, in the model of CD and RA, visual comfort can influence CD and RA to a lower extent than acoustic comfort. For example, the differences in the levels of CD and RA at points 13, 19, 6, 21, and 12 were no more than 0.60, while it reached 1.44 in the model for EF and 1.06 in the model for FD, which testified to the results of stepwise regression models in that acoustic comfort was in a dominant position for CD and RA, and visual comfort was dominant for EF and FD. 

**Figure 4:**
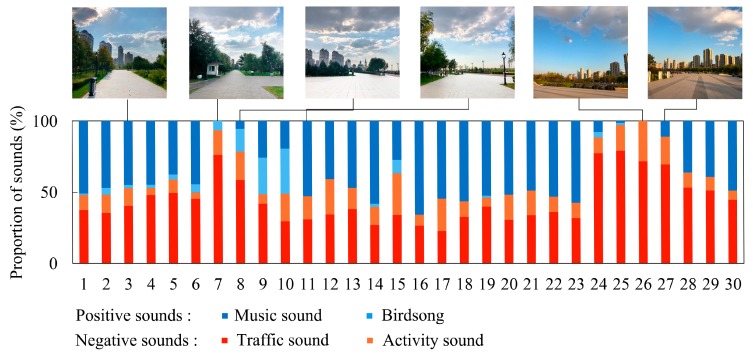
Proportion of sounds in each survey point and photographs in typical survey points in Music Park.

On the other hand, in the AV quadrant, audio-visual context at survey point 11 was most cheerful, relaxed and focused, with the levels of CD, RA and FD were 2.25, 2.25, and 1.56, and point 3 had the most energetic audio-visual context with a value of EF at 2.00. In the UV and UU quadrants, point 7 had the most relaxed, energetic, and focused audio-visual context, with the scores of RA, EF and FD at 1.31, 1.19, and 1.13, respectively. Point 8 had scores for RA, EF, and FD of 1.25, 1.13, and 1.00, respectively. The audio-visual context at point 8 was most cheerful, and the score for CD was just 0.06 higher than that of point 7, although the percentage of negative sounds at points 7 and 8 reached 93.2% and 78.5% (Figure 4), respectively. It is interesting to note that, though the proportion of negative sounds at points 24–27 also were at a higher level, ranging from 88.3%–100%, the audio-visual context at these points was almost the most depressed, anxious, fatigued, and distracted, with the level of psychological responses lower than −1.13. The most likely reason may be that participants can be easily influenced by urban views and sounds due to the lack of a masking effect of vegetation.

## 4. Discussion

Previous studies focused on explaining the relationship between green spaces and psychological responses through visual factors or acoustic factors. Although a few studies emphasized the implications of audio-visual interactions on psychological responses, audio-visual interactions were not considered as two-dimensional. Therefore, this study introduced a new method to quantify audio-visual context, audio-visual experiences, and psychological responses in two typical urban parks in China. The relationship between audio-visual context and psychological responses was studied firstly in this paper, and results show that loudness and sharpness were not correlated with psychological responses of the cognition dimension, which was not inconsistent with Shu and Ma’s study [25]. One possible reason may be that they took children’s psychological responses as a research object. In a recent study in UK, Payne and Bruce reported that relationships between sound levels (subjective and objective) and psychological restoration were not linear, which was also different from this study [16]. It may be because the type of sounds heard and other aspects of the place experience affect the relationships. According to the orthogonal analysis, the psychological responses in audio-visual contexts close to an urban built environment was worse than that in a natural audio-visual context, which was similar to previous related studies [7,8,19].

This study makes a significant contribution to the literature because a new method was designed and executed to specify the audio-visual interactions that affect the psychological responses of young people and how the audio-visual context based on psychological responses relates to the design of soundscapes and landscapes of urban green spaces.

However, there were some limitations to this study. This study employed graduate students as an example of young people, which may have resulted in bias. The psychological state of other young people, such as young workers and officers, was not at the same level as that of graduate students, which may influence the relationship between audio-visual interactions and psychological responses. Therefore, they will be invited to be controlled subjects in future studies. Moreover, a recent work proposed that the differences in seasonal effects may lead to different audio-visual contexts, and psychological responses could consequently be affected [49]. To avoid seasonal influence, this study was carried out only in one season. Furthermore, in the preliminary survey, some participants reported that their emotions could be changed by styles of music in Music Park. To reduce the error, the music in Music Park was set to classical music. The consideration of seasonality, crowd characteristics and types of sounds were important in terms of the creation of audio-visual context in urban green spaces that promotes psychological responses. Thus, in future studies, these factors could also be explained further.

## 5. Conclusions

Based on a new field method—namely, AV-walk—some interesting results in two typical urban parks are summarized below. 

First, on the influence of audio-visual context, there were significant correlations between LAeq and psychological responses and between roughness and psychological responses in Cloves Park (*p* < 0.05), while LAeq and roughness showed no correlation with psychological responses in Music Park.

Second, in terms of the effects of audio-visual experiences, acoustic comfort and visual comfort determined CD, RA, and EF, and only acoustic comfort determined FD in Cloves Park (*p* < 0.01), and the impact intensity of acoustic comfort was 1.55–2.64 times higher than that of visual comfort. In the model for CD and RA (emotion dimension) in Music Park, acoustic comfort was 1.64–1.68 times higher than that of visual comfort, respectively, while in the model for EF and FD (cognition dimension), visual comfort was 1.35–1.37 times than that of acoustic comfort.

In addition, the influence of audio-visual interactions on psychological responses was also analyzed with an orthogonal analysis. The audio-visual context located beside the waterscape with a relatively higher level of acoustic and visual comfort was most cheerful (2.60), relaxed (2.45), and energetic (2.05), and the audio-visual context located on the top of a mountain had better performance; i.e., cheerful (1.50), relaxed (1.30), and energetic (1.35), although the visual context was uncomfortable. The audio-visual context close to an urban built environment, which was both acoustic and visually uncomfortable, was most depressed (−0.25), anxious (−0.75), fatigued (−1.13) and distracted (−1.13).

## Figures and Tables

**Table 1:** The Pearson correlation results between acoustic and visual environments and psychological responses in two parks.

Sig.		Psychological Responses in Cloves Park				Psychological Responses in Music Park			
		CD	RA	EF	FD	CD	RA	EF	FD
Acoustic-visual context	LAeq	0.000 **	0.000 **	0.000 **	0.000 **	0.509	0.370	0.916	0.333
	Loudness	−0.071	0.013 *	0.027 *	0.003 **	0.255	0.455	0.179	0.437
	Roughness	0.030 *	0.004 **	0.009 **	0.001 **	0.436	0.272	0.915	0.289
	Sharpness	0.814	0.731	0.407	0.397	0.961	0.691	0.615	0.673
	Visual green rate	0.774	0.789	0.845	0.738	0.197	0.358	0.451	0.382
	Sky visibility	0.206	0.212	0.174	0.130	0.056	0.046 *	0.183	0.104
Acoustic-visual experience	Acoustic comfort	0.000 **	0.000 **	0.000 **	0.000 **	0.000 **	0.000 **	0.000 **	0.000 **
	Visual comfort	0.000 **	0.000 **	0.001 **	0.004 **	0.000 **	0.000 **	0.000 **	0.000 **

* means *p* < 0.05, ** means *p* < 0.01. cheerful-depressed (CD), relaxed-anxious (RA), energetic-fatigued (EF), focused-distracted (FD). Sig.: significance level.

**Table 2:** Stepwise regression model for audio-visual contexts and psychological responses in Cloves Park.

Model		Collinearity	Unstandardized Coefficients		Standardized Coefficients	Sig. ^a^	Sig. ^b^
Variable	Predictors	VIF	Regression Coefficient	Std. Error	Beta		
CD: *R^2^*= 0.537 *R^2^* (adj) = 0.521	(Constant)		7.415	1.136		0.000 **	0.000 **
	LAeq	1.000	−0.125	0.022	−0.733	0.000 **	
RA: *R^2^*= 0.558 *R^2^* (adj) = 0.542	(Constant)		7.681	1.117		0.000 **	0.000 **
	LAeq	1.000	−0.129	0.022	−0.747	0.000 **	
EF: *R^2^*= 0.533 *R^2^* (adj) = 0.517	(Constant)		7.590	1.231		0.000 **	0.000 **
	LAeq	1.000	−0.135	0.024	−0.730	0.000 **	
FD: *R^2^*= 0.723 *R^2^* (adj) = 0.713	(Constant)		7.095	0.791		0.000 **	0.000 **
	LAeq	1.000	−0.131	0.015	−0.850	0.000 **	

** means *p* < 0.01; ^a^ means significance of regression coefficient; ^b^ means significance of regression equation. VIF: variance inflation factor.

**Table 3:** The stepwise regression model for audio-visual experiences and psychological responses in Cloves Park.

Model		Collinearity	Unstandardized Coefficients		Standardized Coefficients	Sig. ^a^	Sig. ^b^
Variable	Predictors	VIF	Regression Coefficient	Std. Error	Beta		
CD: *R^2^*= 0.870 *R^2^* (adj) = 0.860	(Constant)		0.564	0.096		0.000 **	0.000 **
	Acoustic comfort	1.248	0.400	0.047	0.660	0.000 **	
	Visual comfort	1.248	0.372	0.068	0.427	0.000 **	
RA: *R^2^*= 0.870 *R^2^* (adj) = 0.860	(Constant)		0.779	0.097		0.000 **	0.000 **
	Acoustic comfort	1.248	0.456	0.047	0.748	0.000 **	
	Visual comfort	1.248	0.277	0.068	0.316	0.000 **	
EF: *R^2^*= 0.712 *R^2^* (adj) = 0.691	(Constant)		0.564	0.095		0.000**	0.000 **
	Acoustic comfort	1.001	0.512	0.067	0.783	0.000 **	
	Visual comfort	1.001	0.385	0.134	0.297	0.000 **	
FD: *R^2^*= 0.666 *R^2^* (adj) = 0.654	(Constant)		0.405	0.073		0.000 **	0.000 **
	Acoustic comfort	1.000	0.445	0.059	0.816	0.000 **	

** means *p* < 0.01; ^a^ means significance of regression coefficient; ^b^ means significance of regression equation.

**Table 4:** Stepwise regression model for audio-visual experiences and psychological responses in Music Park.

Model		Collinearity	Unstandardized Coefficients		Standardized Coefficients	Sig. ^a^	Sig. ^b^
Variable	Predictors	VIF	Regression Coefficient	Std. Error	Beta		
CD: *R^2^*= 0.862 *R^2^* (adj) = 0.852	(Constant)		0.557	0.081		0.000 **	0.000 **
	Acoustic comfort	1.512	0.381	0.052	0.649	0.000 **	
	Visual comfort	1.512	0.362	0.082	0.387	0.000 **	
RA: *R^2^*= 0.854 *R^2^* (adj) = 0.843	(Constant)		0.478	0.092		0.000 **	0.000 **
	Acoustic comfort	1.512	0.417	0.059	0.640	0.000 **	
	Visual comfort	1.512	0.405	0.094	0.391	0.000 **	
EF: *R^2^*= 0.612 *R^2^* (adj) = 0.583	(Constant)		−0.167	0.169		0.331	0.000 **
	Visual comfort	1.512	0.595	0.173	0.507	0.002 **	
	Acoustic comfort	1.512	0.273	0.109	0.370	0.018 *	
FD: *R^2^*= 0.758 *R^2^* (adj) = 0.740	(Constant)		−0.280	0.117		0.024 *	0.000 **
	Visual comfort	1.512	0.575	0.119	0.561	0.000 **	
	Acoustic comfort	1.512	0.268	0.075	0.415	0.001 **	

** means *p* < 0.01; ^a^ means significance of regression coefficient; ^b^ means significance of regression equation.
